# Should the Health Community Promote Smokeless Tobacco (Snus): Comments from British American Tobacco

**DOI:** 10.1371/journal.pmed.0040300

**Published:** 2007-10-30

**Authors:** Justine Williamson, Christopher Proctor

Chapman and Freeman question the tobacco industry's aims on snus, and whether these aims can be consistent with harm reduction [1]. We cannot speak on behalf of the industry as a whole. However, we at British American Tobacco understand that cigarette smoking is a major cause of serious and fatal diseases, and we believe that the use of Swedish-style snus products, while not harmless, is substantially less harmful than cigarette smoking [2]. We are piloting snus in several countries outside of Sweden as a response to those public health stakeholders who have told us they believe that snus, properly regulated, can contribute to reducing the net public health impact of tobacco use.

We believe adult consumers of tobacco products would benefit from the enactment of a regulatory framework that facilitates consistent, accurate, and meaningful communications on the relative health risks of smoking, using snus, or abstaining entirely from tobacco use. While ideally this framework would be developed and agreed upon under the Framework Convention for Tobacco Control, the recent second Conference of the Parties to the Framework Convention did not address oral tobacco products and has not assigned a high priority to tobacco product regulation in this area. Given this, we think national governments should develop a regulatory framework for snus. In doing so, we think that governments should be mindful of the concerns expressed by Chapman and Freeman.

We agree with the recently released preliminary report from the European Union's Scientific Committee on Emerging and Newly Identified Health Risks, which states that “the balance of these effects [beneficial versus adverse effects on smoking prevalence] will be highly dependent upon the marketing of the product, the health messages delivered with it, and the extent to which switching to smokeless tobacco products as a harm reduction strategy is endorsed by health professionals and their organisations” [3].

We acknowledge that some have concerns with regard to our interest in snus as a less harmful alternative to cigarettes. We seek to work with the public health community and regulators to achieve a reduction in the public health impact of tobacco use.
